# Suicidal thoughts and behaviors associated with fluoroquinolone antibiotics: a real-world pharmacovigilance analysis

**DOI:** 10.3389/fphar.2025.1556159

**Published:** 2025-04-25

**Authors:** Lijuan Yang, Congqin Chen, Lingqing Ding, Tingting Lu, Xiwen Li, Jie Xiao

**Affiliations:** Department of Pharmacy, Xiamen Cardiovascular Hospital of Xiamen University, School of Medicine, Xiamen University, Xiamen, China

**Keywords:** FAERS, fluoroquinolones, pharmacovigilance, suicidal thoughts, suicidal behaviors

## Abstract

**Objective:**

This study aimed to systematically and scientifically investigate the potential associations between the use of fluoroquinolone antibiotics (ciprofloxacin, levofloxacin, moxifloxacin, ofloxacin, norfloxacin, and delafloxacin) and suicidal thoughts and behaviors using data from the Food and Drug Administration Adverse Event Reporting System (FAERS) database.

**Methods:**

The FAERS database was queried from the first quarter of 2004 to the fourth quarter of 2023. Disproportionality analysis was conducted using the reporting odds ratio (ROR) and empirical Bayes geometric mean (EBGM).

**Results:**

A total of 737 cases of suicidal thoughts and behaviors associated with fluoroquinolones (FQs) were reported in the FAERS database during the study period. Overall, FQs did not demonstrate a disproportionate increase in overall cases of suicidal thoughts and behaviors (ROR: 0.74, 95% CI: 0.69–0.79, P < 0.001; EBGM05: 0.69). Stratified analyses revealed no safety signals for suicidal thoughts and behaviors associated with FQs in either females or males. However, subgroup analyses by age groups demonstrated slightly elevated RORs for suicidal thoughts and behaviors in the <18 years age group (ROR: 1.51, 95% CI: 1.05–2.19, P = 0.03) and the 18–24 years age group (ROR: 2.31, 95% CI: 1.75–3.06, P < 0.001), although the EBGM05s values remained below two in both populations. No significant safety signals were observed in the other age groups.

**Conclusion:**

The analysis of reported cases of suicidal thoughts and behaviors in the FAERS database does not indicate an overall safety signal associated with fluoroquinolones (FQs) at present. Subgroup analysis revealed a slight increase in the RORs for suicidal thoughts and behaviors in the <18 years and the 18–24 years age group; however, no significant safety signal was detected based on the EBGM05s in these populations. Further comprehensive and prospective studies are necessary to confirm and validate these findings.

## 1 Introduction

Fluoroquinolones (FQs) are a group of antibiotics commonly used to treat a variety of bacterial infections, including urinary tract infections, gastrointestinal infections, respiratory tract infections, sexually transmitted diseases, bacterial bronchitis, pneumonia, sinusitis, septicemia, intra-abdominal infections, joint and bone infections and skin infections (Brar et al., 2020; Choi et al., 2023; Silva et al., 2024; Collins et al., 2022). As the first fully synthetic antibiotics, FQs exert their antimicrobial effects by inhibiting DNA gyrase and topoisomerase IV, thereby disrupting bacterial DNA synthesis and ultimately leading to rapid bacterial cell death (Blondeau, 2004). Owing to their favorable pharmacokinetic characteristics, high oral bioavailability, and wide range of antimicrobial effectiveness, fluoroquinolones were the third most frequently prescribed class of antibiotics in the United States in 2011 (Hicks et al., 2015). Despite their clinical efficacy, FQs are associated with a range of well-documented adverse effects on the gastrointestinal, dermatological, cardiovascular, and nervous systems (Rubinstein, 2001; Huruba et al., 2021; Appelbaum and Hunter, 2000). However, the potential impact of FQs on mental health remains insufficiently understood. Among the reported psychiatric side effects of FQs, suicidal thoughts and behaviors have raised significant concerns. Cases of suicidal ideation, suicide attempts, and completed suicides have been reported following the initiation of FQ therapy (Zhang et al., 2021), (Ahmed et al., 2011; Labay-Kamara et al., 2012; Dyer, 2023).

In August and September 2023, the UK Medicines and Healthcare Products Regulatory Agency (MHRA) issued an alert highlighting the psychiatric risks associated with fluoroquinolones (FQs), including ciprofloxacin, delafloxacin, levofloxacin, moxifloxacin, and ofloxacin, which may lead to suicidal thoughts and behaviors (MHRA, 2024; MHRA, 2024). This alert was prompted by a coroner’s report investigating the death of a respected consultant cardiologist who retired in May 2022 at the age of 63 and tragically died by suicide 11 days after initiating ciprofloxacin treatment for prostatitis symptoms. The coroner emphasized the rare but severe side effects of ciprofloxacin, particularly in patients without a prior history of mental health issues (Dyer, 2023; MHRA, 2024). However, the MHRA alert did not specify whether males or females were more susceptible to the psychiatric risks associated with fluoroquinolones, nor did it provide details on the age range of individuals affected by these risks (MHRA, 2024; MHRA, 2024).Consequently, based on the available information, it is not possible to determine which demographic groups are more vulnerable, underscoring the need for further research to clarify these critical aspects and inform targeted interventions. In light of the widespread global use of fluoroquinolones, it is essential to investigate potential relationships between fluoroquinolones and suicidal thoughts and behaviors. This inquiry is highly important for ensuring public safety and optimizing the clinical use of these widely prescribed antibiotics.

## 2 Methods

### 2.1 Data source

The FDA Adverse Event Reporting System (FAERS) is a publicly available database created to support the FDA’s efforts in monitoring the safety of drugs and therapeutic biologic products after they have been marketed (Ding et al., 2022). Reports were evaluated quantitatively through signal detection, with a signal indicating an adverse event (AE) related to a drug. Researchers have started analyzing various drugs and diseases using the FAERS database to identify potential AEs that warrant special attention, with the goal of offering guidance for clinical medication and treatment (Chen et al., 2023a; Chen et al., 2023b). AE symptoms are classified using the internationally recognized and clinically validated Medical Dictionary for Regulatory Activities (MedDRA) terminology (Kumar, 2019). FAERS allows for the analysis of unexpected adverse event trends that may be overlooked in clinical trials owing to limitations in participant diversity (Yu et al., 2021; Xuan et al., 2023).

### 2.2 Data queries

**FIGURE 1:**
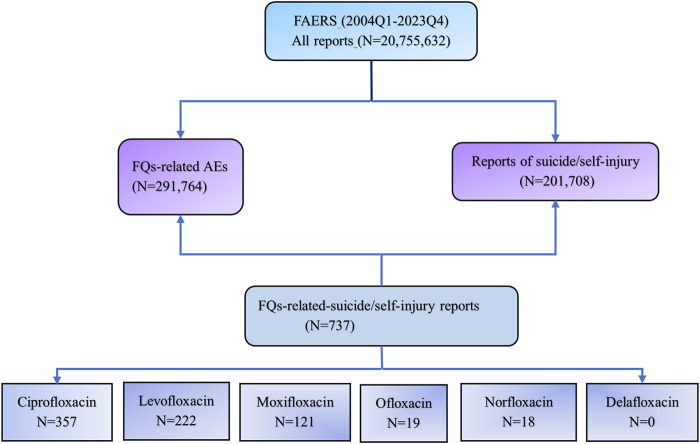
Flow chart of data queries within the FAERS database. FAERS, the U.S. Food and Drug Administration (FDA) Adverse Event Reporting System; Q1, the first quarter; Q4, the fourth quarter; FQs, fluoroquinolones.

The FAERS database was queried from the first quarter (Q1) of 2004 to the fourth quarter (Q4) of 2023 to analyze disproportionality and investigate the associations between FQs and AEs involving suicidal thoughts and behaviors. Each report was categorized according to the contingency table (Table 1), where “a” denotes the count of suicide reports associated with FQs, “b” indicates the count of reports for FQs that do not involve suicide, “c” represents the number of suicide reports related to all other drugs, and “d” signifies the reports for all other drugs that do not involve suicide. Reports related to FQs were identified when any of the six FQs (ciprofloxacin, levofloxacin, moxifloxacin, ofloxacin, norfloxacin, and delafloxacin) were categorized as either “primary suspected” or “secondary suspected” drugs in the reported cases. Instances of suicide and self-injuries were examined using the MedDRA 27.0 preferred terms (PTs) associated with the standard MedDRA query (SMQ) for “suicide/self-injury.” The specific terminology employed included “assisted suicide,” “abnormal Columbia Suicide Severity Rating Scale”, “completed suicide”, “depression with suicidal tendencies”, “intentional overdose”, “deliberate self-injury”, “intentional poisoning”, “self-injurious thoughts”, “suicidal actions”, “suicidal thoughts”, “suicide attempt”, “suicide threat”, “suspected suicide”, and “suspected suicide attempt”. Using universally recognized and clinically validated Medical Subject Headings (MeSH) terms, we were able to address reports relevant to the objectives of this study effectively. The comprehensive data processing procedure is illustrated in Figure 1.

**TABLE 1:** Contingency table for disproportionality analysis.

	Event suicide/self-injury	All other events
FQs	a	b
All other drugs	c	d

Abbreviations: FQs, fluoroquinolones.

### 2.3 Statistical analysis

A population-based pharmacovigilance study using case/noncase methodology was conducted to explore the possible link between FQs and reports of suicidal behavior and/or self-injury. This approach is commonly used in pharmacovigilance research to identify safety signals (Dong and Sun, 2022; Ji et al., 2023; Al-Yafeai et al., 2024). From a mathematical standpoint, the case/noncase methodology involves comparing the incidence rate of a specific AE in patients who have taken a certain medication with the incidence rate of the same AE in patients who have not taken that medication (Rothman et al., 2004; Sakaeda et al., 2013). In this study, we assessed disproportionality using the empirical Bayes geometric mean (EBGM) derived from the multi-item gamma Poisson shrinker (MGPS) and the reporting odds ratio (ROR). A signal was identified when the EBGM05 metric, which represents the lower one-sided 95% confidence limit of the EBGM, was greater than or equal to 2.0 (26) or when the lower limits of the 95% confidence intervals (95% CIs) for the ROR exceeded one in at least three reports (Chen L. et al., 2024). The formulas for calculating the EBGM and ROR are provided in Table 2. All data processing and statistical analyses were performed using SPSS software (version 29.0).

**TABLE 2:** Equations and criteria for ROR and MGPS.

Algorithms	Equation	Criteria
ROR	ROR = ad/b/c	Lower limit of 95% CI > 1, N ≥ 3
	95% CI = e ^ln (ROR)±1.96(1/a+1/b+1/c+1/d) ^0.5^	
MGPS	EBGM = a (a + b + c + d)/(a + c)/(a + b)	EBGM05 > 2
	95% CI = e ^ln (EBGM)±1.96(1/a+1/b+1/c+1/d) ^0.5^	

^a^ the number of suicide/self-injury reports for FQs; b, the reports for FQs, without suicide/self-injury; c, the number of suicide/self-injury reports for all other drugs; d, the reports for all other drugs without suicide/self-injury; 95% CI, 95% confidence interval; N, the number of reports; ROR, the reporting odds ratio; MGPS, multi-item gamma Poisson shrinker; EBGM, empirical Bayesian geometric mean; EBGM05, the lower limit of the 95% CI, of EBGM; FQs, fluoroquinolones.

## 3 Results

### 3.1 General characteristics

During the study period, a total of 20,755,632 unique records were extracted from the FAERS database. Of these, 201,708 AEs were associated with FQs, and 291,764 reports involved suicide or self-injury. Among these, FQs were identified as the suspected drug linked to suicide or self-injury in 737 reports.

The clinical characteristics of the cases involving suicide or self-injury associated with FQs are summarized in Table 3. Among these cases, 53.46% of the individuals were female, whereas 40.57% were male. The majority of reported incidents occurred in adults aged 25–64 years, accounting for 60.52% of the total cases. Reports were predominantly submitted by consumers (46.40%), followed by physicians (20.22%), pharmacists (8.41%), and other healthcare providers (12.21%). Notably, AEs related to suicide or self-injury associated with FQs were most frequently recorded in 2023. A detailed review of all reported cases revealed that 57.53% exhibited symptoms consistent with suicidal behavior, 13.70% resulted in completed suicide, and 11.40% involved suicide attempts.

**TABLE 3:** Clinical features of suicide/self-injury cases associated with FQs reported in the FAERS during the study period.

Categories	Ciprofloxacin *N* (%)	Levofloxacin *N* (%)	Moxifloxacin *N* (%)	Ofloxacin *N* (%)	Norfloxacin *N* (%)	Delafloxacin *N* (%)	Total *N* (%)
Reports of FQs	357	222	121	19	18	0	737
Age group, (y)							
<18	17 (4.76)	5 (2.25)	0 (0.00)	0 (0.00)	7 (38.89)	—	29 (3.93)
18–24	31 (8.68)	9 (4.05)	7 (5.79)	1 (5.26)	3 (16.67)	—	51 (6.92)
25–64	217 (60.78)	135 (60.81)	72 (59.50)	16 (84.21)	6 (33.33)	—	446 (60.52)
>64	38 (10.64)	28 (12.61)	9 (7.44)	0 (0.00)	1 (5.56)	—	76 (10.31)
Unknown or missing	54 (15.13)	45 (20.27)	33 (27.27)	2 (10.53)	1 (5.56)	—	135 (18.32)
Median (IQR)	46.00 (32.00–60.00)	43.00 (35.00–7.00)	44.00 (33.00–60.75)	49.00 (40.50–55.00)	19.00 (17.00–47.50)	—	44.50 (32.00–59.00)
Gender							
Male	158 (44.26)	91 (40.99)	36 (29.75)	13 (68.42)	1 (5.56)	—	299 (40.57)
Female	186 (52.10)	113 (50.90)	76 (62.81)	4 (21.05)	15 (83.33)	—	394 (53.46)
Unknown or missing	13 (3.64)	18 (8.11)	9 (7.44)	2 (10.53)	2 (11.11)	—	44 (5.97)
Reporter							
Physician	71 (19.89)	34 (15.32)	33 (27.27)	5 (26.32)	6 (33.33)	—	149 (20.22)
Pharmacist	25 (7.00)	14 (6.30)	15 (12.40)	1 (5.26)	7 (38.89)	—	62 (8.41)
Other health- professional	44 (12.32)	18 (8.11)	17 (14.05)	8 (42.11)	3 (16.67)	—	90 (12.21)
Lawyer	6 (1.68)	1 (0.45)	0 (0.00)	0 (0.00)	0 (0.00)	—	7 (0.95)
Consumer	163 (45.66)	130 (58.56)	44 (36.36)	4 (21.05)	1 (5.56)	—	342 (46.40)
Unknown or missing	48 (13.45)	25 (11.26)	12 (9.92)	1 (5.26)	1 (5.56)	—	87 (11.80)
Reporting year							
2004	6 (1.68)	4 (1.80)	3 (2.48)	0 (0.00)	2 (11.11)	—	15 (2.04)
2005	5 (1.40)	8 (3.60)	9 (7.44)	0 (0.00)	0 (0.00)	—	22 (2.99)
2006	17 (4.76)	11 (4.95)	1 (0.83)	1 (5.26)	0 (0.00)	—	30 (4.07)
2007	7 (1.96)	12 (5.41)	5 (4.13)	0 (0.00)	0 (0.00)	—	24 (3.26)
2008	13 (3.64)	14 (6.31)	12 (9.92)	0 (0.00)	0 (0.00)	—	39 (5.29)
2009	8 (2.24)	8 (3.60)	11 (9.09)	0 (0.00)	2 (11.11)	—	29 (3.93)
2010	11 (3.08)	20 (9.01)	7 (5.79)	1 (5.26)	1 (5.56)	—	40 (5.43)
2011	15 (4.20)	11 (4.95)	12 (9.92)	1 (5.26)	1 (5.56)	—	40 (5.43)
2012	34 (9.52)	16 (7.21)	4 (3.31)	7 (36.84)	2 (11.11)	—	63 (8.55)
2013	42 (11.76)	9 (4.05)	0 (0.00)	4 (21.05)	0 (0.00)	—	55 (7.46)
2014	12 (3.36)	11 (4.95)	2 (1.65)	0 (0.00)	1 (5.56)	—	26 (3.53)
2015	16 (4.48)	10 (4.50)	5 (4.13)	0 (0.00)	0 (0.00)	—	31 (4.21)
2016	11 (3.08)	13 (5.86)	2 (1.65)	0 (0.00)	1 (5.56)	—	27 (3.66)
2017	5 (1.40)	5 (2.25)	1 (0.83)	0 (0.00)	0 (0.00)	—	11 (1.49)
2018	7 (1.96)	6 (2.70)	2 (1.65)	0 (0.00)	0 (0.00)	—	15 (2.04)
2019	10 (2.80)	6 (2.70)	11 (9.09)	0 (0.00)	1 (5.56)	—	28 (3.80)
2020	7 (1.96)	2 (0.90)	4 (3.31)	0 (0.00)	0 (0.00)	—	13 (1.76)
2021	19 (5.32)	7 (3.15)	9 (7.44)	0 (0.00)	0 (0.00)	—	35 (4.75)
2022	41 (11.48)	14 (6.31)	13 (10.74)	1 (5.26)	0 (0.00)	—	69 (9.36)
2023	71 (19.89)	35 (15.77)	8 (6.61)	4 (21.05)	7 (38.89)	—	125 (16.96)

IQR, interquartile range; FQs, fluoroquinolones.

### 3.2 Signal detection

**FIGURE 2:**
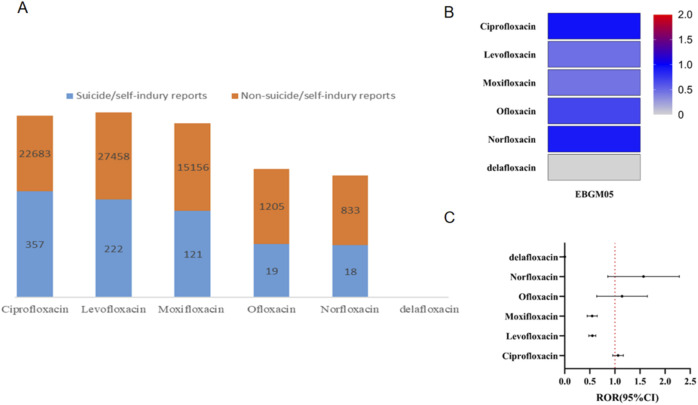
Results of disproportionality analysis for suicidal and self-injurious reports associated with FQs at the drug level. **(A)** Number of suicide/self-injury and nonsuicide/self-injury reports for each FQ drug. **(B)** EBGM05 of FQ-associated suicide/self-injury for each distinct FQ drug. **(C)** RORs (95% CI) of FQ-associated suicide/self-injury for each distinct FQ drug. ROR, reporting odds ratio; FQ, fluoroquinolone; EBGM05, lower one-sided 95% confidence limit (95% CI) of the empirical Bayes geometric mean.

Figure 2A illustrates the number of reports associated with each specific FQ drug. Among the reports of suicidal and self-injurious behavior related to FQs, ciprofloxacin accounted for 357 cases, levofloxacin accounted for 222 cases, moxifloxacin accounted for 121 cases, ofloxacin accounted for 19 cases, and norfloxacin accounted for 18 cases. No reports were linked to delafloxacin. Further stratified analyses revealed low ROR and EBGM05 values for suicide/self-injury across all FQs, suggesting that none of the six FQ drugs investigated in this study were associated with an increased risk of suicide/self-injury overall (Figures 2B, C).

**FIGURE 3:**
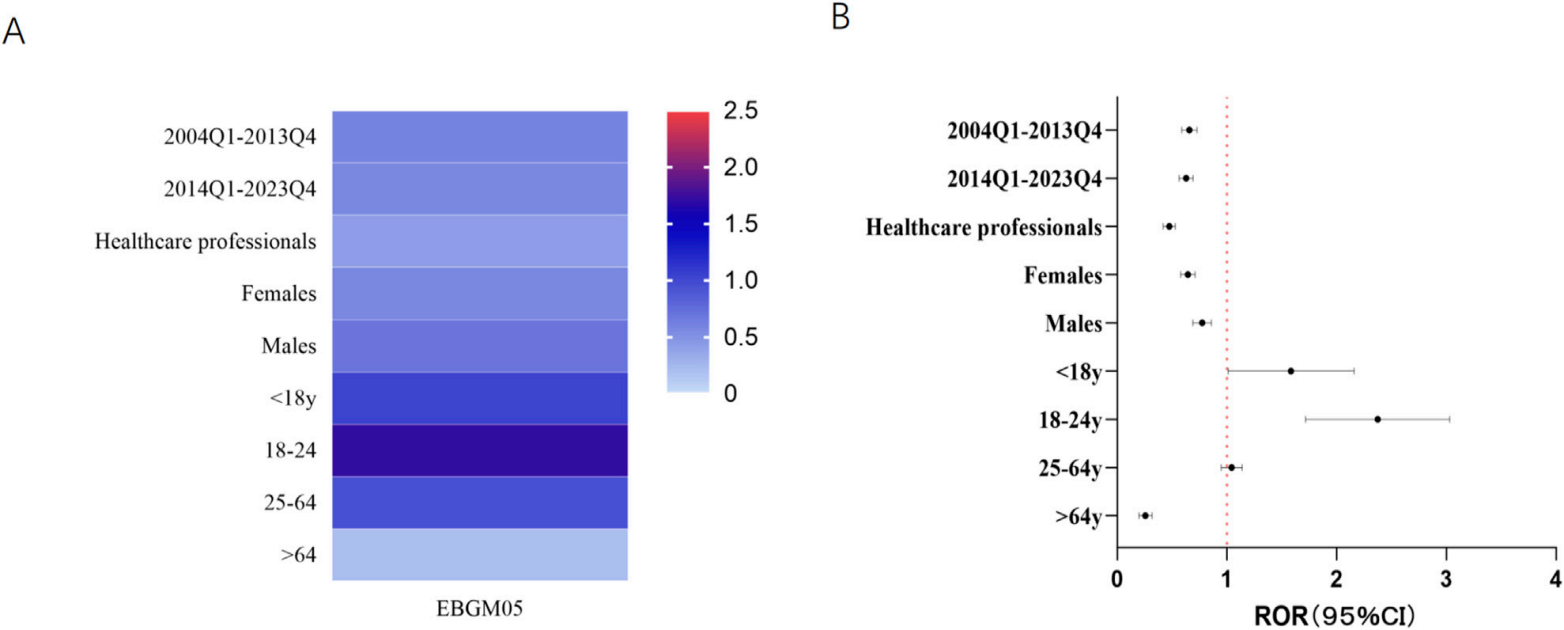
Results of subgroup disproportionality analysis of FQs associated with suicide/self-injury on the basis of age, sex and reporting time. **(A)** EBGM05s of FQ-associated suicide/self-injury in different sex, age, reporter-type and reporting time groups. **(B)** RORs (95% CI) of FQ-associated suicide/self-injury in different sex, age, reporter-type and reporting time groups. ROR, reporting odds ratio; 95% CI, 95% confidence limit; FQs, fluoroquinolones; EBGM05, lower one-sided 95% CI of the empirical Bayes geometric mean.

To explore individual characteristics more thoroughly, separate subanalyses were performed on the basis of sex and age (Figure 3). In both females (ROR 0.64, 95% CI 0.59–0.71, P < 0.001; EBGM05 0.58) and males (ROR 0.77, 95% CI 0.69–0.86, P < 0.001; EBGM05 0.69), no safety signal indicating suicide/self-injury associated with FQs was identified. Stratified analysis based on age revealed that the RORs for suicide/self-injury associated with FQs was slightly elevated in the <18 years age group (ROR: 1.51, 95% CI: 1.05–2.19, P = 0.028) and the 18–24 years age group (ROR: 2.31, 95% CI: 1.75–3.06, P < 0.001). However, the EBGM05s values remained below two in these populations. No significant signals were observed in the other age groups.

## 4 Discussion

The neuropsychiatric safety of fluoroquinolones remains a pressing area of investigation, given their extensive global use and potential to adversely affect mental health. This concern has been underscored by the MHRA alert, which highlights reports of suicidal thoughts and behaviors associated with FQ use. In response, we conducted a comprehensive analysis to evaluate the potential risk of suicide linked to various FQs.

The central nervous system (CNS) toxicity of quinolone antibiotics is well documented and manifests as a range of symptoms, such as hallucinations, dizziness, and headaches. Several potential neurobiological mechanisms have been proposed to explain the associations between fluoroquinolones and CNS events (Wierzbiński et al., 2023; Scavone et al., 2020; Wang et al., 2022). Owing to the structural resemblance of fluoroquinolones to γ-aminobutyric acid (GABA) agonists, fluoroquinolones may interact with GABA receptors in the brain, potentially resulting in neurotoxicity (Wierzbiński et al., 2023; Scavone et al., 2020; Wang et al., 2022). It has been proposed that FQs might activate excitatory N-methyl-d-aspartate (NMDA) and adenosine receptors, and this could lead to noticeable CNS symptoms if FQs penetrate the CNS sufficiently (Wierzbiński et al., 2023; Wang et al., 2022). Some researchers suggest that fluoroquinolones may contribute to neuro-AEs by inducing a reduction in serotonin levels, increasing oxidative stress, lowering antioxidant levels, and altering various microRNAs (Wang et al., 2022). However, whether these mechanisms contribute to suicidal thoughts or self-injurious behaviors remains a matter of debate, and further research is needed.

The existing evidence regarding the association between FQs and the risk of suicidal or self-injurious behavior remains limited. Wang and colleagues [30] conducted a nationwide cohort study involving over one million U.S. patients treated with fluoroquinolones for pneumonia or urinary tract infections (UTIs). Their analysis revealed no increased risk of suicidality associated with short-term use of fluoroquinolones compared with azithromycin or trimethoprim-sulfamethoxazole, with adjusted hazard ratios of 1.01 (95% confidence interval: 0.76–1.36) for the pneumonia cohort and 1.03 (95% confidence interval: 0.91–1.17) for the UTI cohort. Similarly, a nested case‒control study by Jick et al. involving 348 individuals with suicidal ideation, attempts, or completed suicides and 808 controls reported no significant association between quinolone antibiotics and an increased risk of suicidal behaviors. Our study aligns with these findings, as subgroup analyses revealed no safety signals related to suicide or self-injury for any of the six fluoroquinolones investigated. This finding is consistent with previous research and further supports the conclusion that fluoroquinolones, as a class, are not associated with an increased risk of suicidal thoughts or behaviors. Additionally, the subgroup analysis enhances our understanding of the safety profiles of individual FQ drugs in relation to these neuropsychiatric outcomes, reinforcing their clinical applicability under appropriate use.

We identified 737 cases of suicidal and self-injurious behavior associated with FQs in the FAERS database from 2004Q1 to 2023Q4. Among these patients, 53.46% were female, and 40.57% were male. Epidemiological data on suicide suggest that the 12-month prevalence of suicidal thoughts is generally higher in females than in males, whereas suicide attempt rates are comparable between the sexes (Borges et al., 2010). In our study, 424 cases (57.53%) involved reports of suicidal ideation, potentially explaining the greater proportion of females in this group. Subgroup disproportionality analysis by sex revealed no significant safety signals for either females or males, indicating no notable differences in the incidence of these adverse events, which requires further investigation.

For adolescents and young adults, suicide continues to be a leading cause of mortality worldwide and is a significant public health issue (McLoughlin et al., 2015; Bertuccio et al., 2024; Hutchinson et al., 2025). Suicide is the second leading cause of mortality among individuals aged 10–24 in the United States (US) (Graham et al., 2025). According to the Centers for Disease Control and Prevention, suicidality and suicidal behavior among youth continue to increase significantly each year (Scudder et al., 2022). The suicide rates among youth have risen precipitously over the past 3 decades with a 52.2% increase between 2000 and 2021 (35). We performed a subgroup analysis by age to evaluate the proportional imbalance of adverse events related to suicide and self-injury associated with FQs across various age groups. In particular, among children and young adults, the RORs showed a slight increase in the reporting of suicide and self-injury. However, it is important to interpret these results cautiously, as the EBGM05 values for fluoroquinolone-associated suicide and self-injury in children and young adults were less than 2, indicating a lack of strong signal strength. Although the stratified analyses were based on a limited number of exposed patients, the elevated RORs for suicidal and self-injurious behavior in the <18 years age group and 18–24 years age group suggest that young patients may be at increased risk during fluoroquinolone use.

Our study had several limitations. First, the majority of reports lacked evidence to establish a definitive causal relationship between the reported AEs and fluoroquinolone exposure. The inability to conclusively determine causality is a limitation that is prevalent in all pharmacovigilance studies (Chen J. et al., 2024; Zhao et al., 2024; Cho et al., 2024). Second, cases in the FAERS database often contain incomplete information, such as details on dosage, comorbidities, time to onset, and other relevant factors. This lack of comprehensive data limits the ability to assess a drug’s safety profile completely. As a result, our study cannot provide a complete evaluation of the relationship between FQ dosages or indications and the risk of suicide or self-injury. Additionally, this study does not have the capacity to assess other potential risk factors or comorbid conditions thoroughly. To address these challenges, we conducted a detailed stratified analysis to explore potential factors influencing the neuropsychiatric safety of fluoroquinolones. The risk of neuropsychiatric adverse events may be associated with multiple variables, including gender, age, reporting year, and reporter type. This study is a pharmacovigilance analysis based on an adverse event reporting database, aiming to detect post-marketing safety signals. The fundamental principle of disproportionality analysis relies on the imbalance in reporting proportions, which can be extracted from real-world data. In our study, beyond assessing the overall risk, we performed stratified analyses based on gender, age, and reporting year to determine whether the risk is elevated under specific conditions and to assess the influence of these factors. Furthermore, to improve the reliability of our findings and minimize confounding biases, we conducted a secondary analysis (Model 2), which included only adverse event reports submitted by healthcare professionals. Reports from healthcare professionals generally undergo a more rigorous causality assessment, enhancing reliability and potentially reducing confounding influences. By incorporating these analytical strategies, we aimed to refine the interpretation of fluoroquinolone-associated neuropsychiatric risks while acknowledging the inherent limitations of spontaneous reporting data. Consequently, any conclusions derived from the pharmacovigilance analysis should be understood in light of these limitations, and additional research with a wider focus may be needed to provide a more comprehensive understanding of the relationships involved. Despite these limitations, disproportionality analysis continues to be an essential tool for identifying potential safety signals related to medications and directing further investigations (Yu et al., 2021; Nair et al., 2023). Importantly, our study provides an initial overview on the basis of the data and methodologies currently available. This finding requires validation through additional clinical studies.

## 5 Conclusion

Concerns regarding the potential risk of suicide associated with FQs have primarily stemmed from anecdotal case reports. This study contributes postmarket evidence to the understanding of the neuropsychiatric safety profile of FQs. Our analysis of suicide and self-injury cases reported in the FAERS database does not indicate any significant safety signals directly linked to FQs. However, it is essential to recognize that this study provides a preliminary assessment on the basis of the limitations of the available data and methodologies. To confirm these findings, larger-scale and more comprehensive prospective studies are urgently needed.

## Data Availability

The datasets presented in this study can be accessed online. The names of the repository/repositories and accession number(s) can be found below: https://www.fda.gov/drugs/questions-and-answers-fdasadverse-event-reporting-system-faers/fda-adverse-event-reportingsystem-faers-public-dashboard.
